# Predictors of the Need for an Atrial Septal Defect Closure at Very Young Age

**DOI:** 10.3389/fcvm.2019.00185

**Published:** 2020-01-10

**Authors:** Gustaf Tanghöj, Petru Liuba, Gunnar Sjöberg, Estelle Naumburg

**Affiliations:** ^1^Department of Clinical Sciences, Unit of Pediatrics, Umeå University, Umeå, Sweden; ^2^Department of Cardiology, Pediatric Heart Center, Skåne University Hospital, Lund, Sweden; ^3^Department of Women's and Children's Health, Karolinska Institute, Stockholm, Sweden

**Keywords:** atrial septal defect, ASD, follow-up studies, heart septal defect, atrial, pediatric cardiology, pediatric thoracic surgery, septal device occlusion

## Abstract

An asymptomatic Atrial Septal Defect (ASD) is often closed at the age of 3–5 years using a transcatheter or surgical technique. Symptomatic ASD or ASD associated with pulmonary hypertension (PHT) may require earlier closure, particularly in combination with other non-cardiac risk factors for PHT, but the indications for early closure and the potential risk for complications are largely unknown. The aim of this study was to assess risk factors for needing ASD closure during the first and second years of life. This case-control study included all children treated with surgical or percutaneous ASD closure between 2000 and 2014 at two out of three pediatric heart centers in Sweden. “Cases” were children with ASD closure at ≤1 or ≤2 years of age. Clinical data were retrieved from medical journals and national registries. Overall, 413 children were included. Of these, 131 (32%) were ≤2 years, and 50 (12%) were ≤1 year. Risk factors associated with a ≤2 years ASD closure were preterm birth, OR = 2.4 (95% CI: 1.5–3.9); additional chromosomal abnormalities, OR = 3.4 (95% CI: 1.8–6.5); pulmonary hypertension, OR = 5.8 (95% CI: 2.6–12.6); and additional congenital heart defects, OR = 2.6 (95% CI: 1.7–4.1). These risk associations remained after adjustments for confounding factors, such as need for neonatal respiratory support, neonatal pulmonary diseases, neonatal sepsis, additional congenital heart defects (CHD) and chromosomal abnormalities. ASD size:body weight ratio of 2.0, as well as a ratio of 0.8 (upper and lower limit of the ASD size:body weight ratios), was associated with increased risk of an early ASD closure. Risk factors such as very premature birth, very low birth weight, congenital, and chromosomal abnormalities, neonatal pulmonary disease and need for ventilation support, as well as pulmonary hypertension, were associated with very early (<1 year of age) ASD closure. Several independent neonatal risk factors were associated with an increased risk of early ASD closure at 2 and at 1 year of age. An ASD size:body weight ratio is a poor predictor for indications for ASD closure.

## Introduction

Asymptomatic Atrial Septal Defects (ASD) are preferably closed when the children have reached the age of 3–5 years (1, 2). A significant ASD causes a left to right shunt leading to volume overload, enlargement of the right atrium and ventricle and altered myocardial structure and function (3). Preterm children may be three times as common among children with percutaneous device closure than in the general Swedish population (4).

The myocardium of the preterm child has irreversible morphological and global structural alterations (5–8). Even long after the neonatal period and into adulthood, the morphology of both the ventricles as well as the function is impaired or altered (6–10). Being born prematurely, prior to 37 gestational weeks, accounts for 6% of all newborns in Sweden (11). Advances in perinatal care over the last 30 years have led to significant improvements in survival rates, but the risk of early death and pulmonary, neurological, cognitive and cardiovascular morbidity remains (12).

We hypothesize that preterm birth is a risk factor for an early ASD closure (surgical or percutaneous device closure) compared to term children, due to the complex comorbidity, often large ASD size in relation to low bodyweight and cardiac alterations. The aim of this study was to assess risk factors for an early ASD closure, taking into account several potential confounding factors, including the ASD size: bodyweight ratio.

## Materials and Methods

### Material

The study included all children born in Sweden who were treated with an ASD closure, surgically or percutaneously, before the age of 18, between January 2000 and December 2014 at Skåne University Hospital in Lund and the Astrid Lindgren Children's Hospital at Karolinska University Hospital in Stockholm, Sweden. This cohort was studied in a previous study on adverse events after ASD closure (13). Cases were children aged 2 years or younger at the time of ASD closure. Controls were children of an older age.

In a second analysis, cases were defined as children of 1 year of age and younger and controls were children older than 1 year of age at the time of ASD closure.

### Exposure Information and Risk Factors

Data were retrieved from medical records and the Swedish National Birth Register (MFR) (14). Gestational age was estimated from the antenatal determination of gestational age by ultrasound and retrieved from MFR. Premature birth was considered to be birth prior to 37 completed gestational weeks and was stratified according to gestational age at birth: late, 32 to <37 weeks; very premature, 28 to < 32 weeks; and extremely premature, <28 weeks. Analyses were also made for children born prior to 32 completed gestational weeks as a group (i.e., very and extremely premature children).

The largest diameter of the ASD was measured using transesophageal echocardiography (TEE) images, expressed in millimeters and retrieved from stored videotapes and digital examinations. A ratio for ASD size (mm):bodyweight (kg) was calculated and the upper and lower limit of the standard deviation distribution, (0.8–2.0), was used for cut-off limits in the multivariate model.

Potential risk factors, such as the need for neonatal respiratory support, neonatal pulmonary diseases, neonatal sepsis, additional congenital heart defects (CHD) and chromosomal abnormalities, were retrieved from MFR and medical records. Factors such as symptoms from volume overload or pulmonary hypertension prior ASD closure were retrieved from medical records.

### Statistical Analyses

Depending on the type of data, mean (std.) or percentage (%) was calculated. Student's *t*-test was used for parametrically distributed variables (unpaired two-sided) and Person's χ^2^ for categorical data. A *p* < 0.05 was considered significant.

Conditional logistic regression was performed to evaluate the association between ASD closure at an age of 2 years or younger and all significant potential risk factors (*p* ≤ 0.05) with a substantial number of exposed cases and controls. Maximum-likelihood estimates of the odds ratio (OR) and 95% confidence interval (CI) were obtained, taking into account potential confounding factors. Regression models were not used to analyze ASD closure in children below 1 year of age, due to small numbers.

Univariate and multivariate conditional logistic regression was performed for early ASD closure for three groups:

- All types of methods for ASD closure
- Percutaneous device closure
- Surgical closure

The IBM SPSS Statistics, Version 25 software (IBM Corporation, New York, USA) was used.

## Results

### Study Population

**Figure 1:**
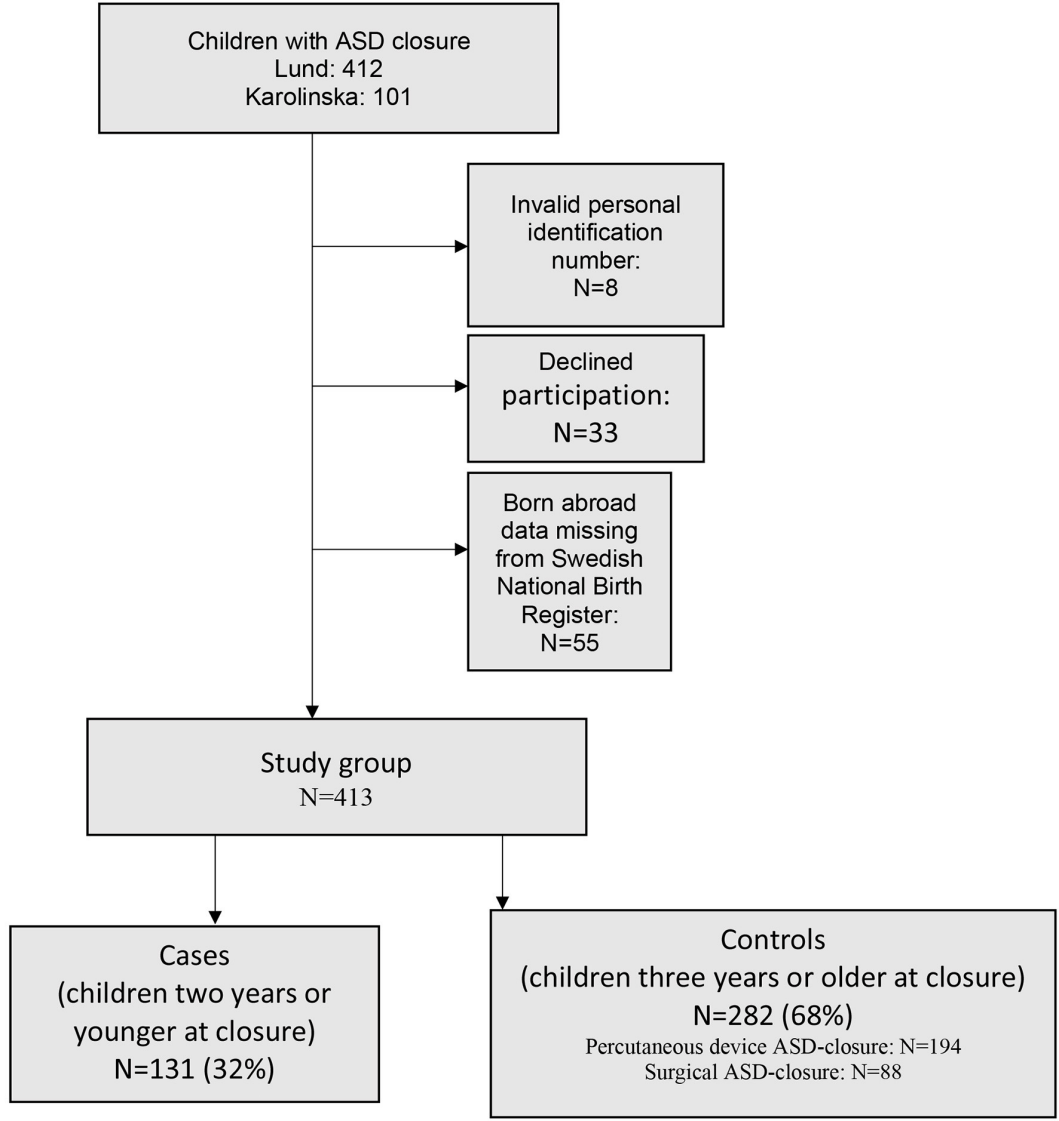
Study-group.

Of a total of 513 children treated with an ASD closure, 98 were excluded due to invalid identification number (*n* = 8), being born abroad (*n* = 55), or due to refusing consent to participate (*n* = 33). Thus, 413 children were included in the study population for analysis (Figure 1).

A total of 131 (32%) cases were 2 years of age or younger at the time of ASD closure. Device closure was more common than surgical closure among controls (*n* = 194 vs. *n* = 88), while type of closure was equally distributed among cases (*n* = 68 vs. *n* = 63). Cases below 2 years of age were born at an earlier gestational age, with a lighter birth weight, and more commonly with comorbidities, such as chromosomal abnormalities, other types of CHD, and required neonatal respiratory support (Table 1).

**Table 1:** Demographic data for ASD closure at 2 years of age or younger.

	**Percutaneous device ASD-closure**			**Surgical ASD-closure**			**All types of ASD closure**		
	**Children <2 years**	**Children >2 years**	***P-value***	**Children <2 years**	**Children >2 years**	***P-value***	**Children <2 years**	**Children >2 years**	***P-value***
Total number	68	194		63	88		131	282	
Weight at closure (kg)	9.6 (±2.4)	24.3 (±16.0)	<0.001	8.1 (±2.5)	21.0 (±11.8)	<0.001	8.9 (±2.5)	23.3 (±14.8)	<0.001
Age at closure (years)	1.3 (±0.4)	6.2 (±1.0)	<0.001	1.0 (±0.5)	5.6 (±3.3)	<0.001	1.2 (±0.5)	6.0 (±3.8)	<0.001
Gestational age (weeks)	36.9 (±4.0)	38.0 (±3.1)	0.021	36.5 (±4.3)	38.1 (±3.6)	0.018	36.7 (±4.5)	38.0 (±3.2)	0.002
ASD size (mm)	11.4 (±3.3)	13.7 (±5.0	0.001	13.0 (±4.2)	14.7 (±6.5)	0.07	12.1 (±3.8)	14.9 (±5.5)	0.001
ASD size:body weight	1.2 (±0.4)	0.7 (±0.4)	<0.001	1.7 (±0.7)	0.8 (±0.4)	<0.001	1.4 (±0.6)	0.7 (±0.4)	0.0001
ASD size:body weight = 2	6 (9%)	1 (1%)	0.001	17 (27%)	1 (1%)	<0.001	23 (17%)	2 (1%)	<0.001
ASD size:body weight = 0.8	56 (82%)	60 (30%)	<0.001	52 (60%)	39 (44%)	<0.001	108 (82%)	99 (35%)	<0.001
Birthweight (gram)	2,821.9 (±902.0)	3,148.5 (±785.6)	0.006	2,695.5 (±896.5)	3,226.0 (±822.0)	<0.001	2,762.7 (±898.2)	3,179.5 (±796.3)	<0.001
Gender (girls/boys)	45/23	116/78	0.135	32/31	55/33	0.151	77/52	171/111	0.328
Preterm birth <37 gestational weeks	23 (39%)	45 (22%)	0.010	21 (33%)	13 (14.8%)	0.007	44 (34%)	49 (17%)	<0.001
Late preterm birth: 32–37 GW	15 (22%)	27 (14%)	0.115	12 (19%)	9 (10%)	0.122	27 (21%)	36 (13%)	0.039
Very preterm birth: 28–32 GW	6 (9%)	4 (2%)	0.012	5 (8%)	0 (0%)	0.011	11 (8%)	4 (1%)	<0.001
Extremely preterm birth: <28 GW	2 (3%)	5 (3%)	0.837	4 (6%)	4 (4%)	0.626	9 (3%)	6 (5%)	0.483
Preterm birth: <32 gestational weeks	8 (12%)	9 (5%)	0.040	10 (16%)	6 (7%)	0.075	20 (15%)	16 (6%)	0.001
Very low birth weight	7 (10%)	9 (5%)	0.094	7 (12%)	5 (6%)	0.217	14 (11%)	14 (5%)	0.039
Extremely low birth weight	3 (4%)	4 (2%)	0.301	4 (7%)	4 (5%)	0.616	7 (6%)	8 (3%)	0.198
Chromosomal abnormalities	15 (22%)	14 (7%)	<0.001	11 (18%)	5 (6%)	0.020	26 (20%)	19 (7%)	<0.001
Other congenital heart defects	17 (25%)	43 (22%)	0.632	36% (57%)	15 (17%)	<0.001	53 (41%)	58 (21%)	<0.001
Arrhythmias prior to closure	3 (4%)	5 (3%)	0.437	2 (3%)	5 (6%)	0.470	5 (4%)	10 (4%)	0.884
Infant respiratory distress syndrome	6 (9%)	12 (6%)	0.459	6 (10%)	2 (2%)	0.069	12 (9%)	14 (5%)	0.104
Bronchopulmonary dysplasia	3 (4%)	7 (4%)	0.766	5 (8%)	2 (2%)	0.131	8 (6%)	9 (3%)	0.168
Neonatal ventilator support	6 (9%)	5 (3%)	0.029	5 (8%)	5 (6%)	0.596	11 (8%)	10 (4%)	0.040
Neonatal CPAP^*^	12 (18%)	17 (9%)	0.048	15 (24%)	6 (7%)	0.003	27 (21%)	23 (8%)	<0.001
Neonatal sepsis	7 (10%)	8 (4%)	0.061	7 (11%)	2 (2%)	0.033	14 (11%)	10 (4%)	0.004
Neonatal pulmonary hypertension	12 (18%)	8 (4%)	<0.001	11 (18%)	2 (2%)	0.001	23 (18%)	10 (4%)	<0.001
Symptomatic ASD	39 (30%)	9 (3%)	<0.001	26 (41%)	2 (2%)	<0.001	13 (19%)	7 (3%)	<0.001
Right ventricular or atrial enlargement as indication for closure							90 (74%)	212 (79%)	0.216
QP:QS > 1.5 as indication for closure							26 (21%)	52 (20%)	0.659
Pulmonary hypertension as indication for closure							14 (12%)	4 (2%)	<0.001

* *Continuous Positive Airway Pressure*.

A large ASD size in relation to bodyweight (ASD size:bodyweight = 2), as well as a smaller ASD size in relation to bodyweight (ASD size:bodyweight = 0.8) was more common among cases of 2 years of age or younger (Table 1).

Pulmonary hypertension being stated as the indication for closure was more common among cases aged 2 years or younger (Table 1).

Overall, 50 (12%) cases were 1 year of age or younger at the time of ASD closure and, of these, 32% had an ASD device closure and 68% a surgical closure (Table 2). Most neonatal and pre-interventional factors were more common among these young cases (Table 2).

**Table 2:** Demographic information on children with ASD closure at 1 year of age or less.

	**Percutaneous device ASD closure**	**Surgical ASD closure**	**All types of ASD closure**		
	**Cases**	**Cases**	**Cases**	**Controls**	***p*-value**
Total number	16 (32%)	34 (68%)	50	363	<0.001
Weight at closure (kg)	6.8 (±1.7)	6.6 (±1.8)	6.6 (±1.7)	20.3 (±14.1)	<0.001
Age at closure (years)	0.7 (±0.2)	0.6 (±0.2)	0.7 (±0.2)	4.9 (±3.8)	<0.001
Gestational age (weeks)	34.0 (±5.5)	35.5 (±5.0)	35.0 (±5.2)	38.0 (±3.2)	<0.001
Birthweight (gram)	1,652.3 (±1070.7)	2,525.5 (±927.0)	2,427.8 (±975.7)	3,138.3 (±796.2)	0.591
Sex (girls/boys)	10/6	13/17	27/23	221/141	<0.001
ASD size	11.1 (±3.2)	12.7 (±3.9)	12.2 (±3.8)	13.6 (±4.2)	0.09
Mean ASD size:bodyweight	1.7 (±0.5)	2.0 (±0.7)	1.9 (±0.7)	0.8 (±0.4)	<0.001
Preterm	8 (50%)	14 (41%)	22 (44%)	71 (20%)	0.876
Late preterm	2 (13%)	6 (18%)	8 (16%)	55 (15%)	<0.001
Very preterm	4 (25%)	4 (12%)	8 (16%)	7 (2%)	0.001
Extremely preterm: <28 gestational weeks	2 (13%)	4 (12%)	6 (12%)	9 (3%)	<0.001
Preterm birth: <32 gestational weeks	6 (37%)	8 (24%)	15 (30%)	21 (6%)	<0.001
Very low birthweight	6 (37%)	5 (15%)	11 (22%)	17 (5%)	0.001
Extremely low birthweight	2 (13%)	4 (12%)	6 (12%)	9 (3%)	<0.001
Chromosomal abnormalities	5 (31%)	8 (23%)	12 (26%)	32 (9%)	<0.001
Other congenital heart defects	7 (44%)	22 (65%)	29 (58%)	82 (23%)	0.351
Arrhythmias before closure	1 (6%)	2 (6%)	3 (6%)	12 (3%)	<0.001
Infant respiratory distress syndrome	4 (25%)	5 (15%)	9 (18%)	17 (5%)	0.003
Bronchopulmonary dysplasia	2 (13%)	4 (12%)	6 (12%)	11 (3.0%)	<0.001
Neonatal ventilator support	4 (25%)	5 (15%)	9 (18%)	12 (3%)	<0.001
Neonatal CPAP^*^	6 (37%)	13 (39%)	19 (38%)	31 (9%)	<0.001
Neonatal sepsis	4 (25%)	6 (18%)	10 (20%)	14 (4%)	<0.001
Pulmonary hypertension	7 (44%)	10 (29%)	17 (34%)	16 (4%)	<0.001
Symptomatic ASD	5 (31%)	20 (59%)	25 (50%)	23 (6%)	<0.001^*^

* *Continuous Positive Airway Pressure*.

### Risk Factors

Being born prematurely was associated with the risk of an ASD closure at 2 years of age and younger, OR = 2.4 (95% CI: 1.5–3.9) for all used ASD closure methods, and even more for surgical ASD closure, OR = 2.9 (95% CI: 1.3–6.3) (Table 3). Severe neonatal morbidities, such as sepsis and the need for neonatal ventilation support of any kind, were associated with increased risk of an early ASD closure, and especially for surgical closure (Table 3).

**Table 3:** Risk factors for ASD closure at 2 years of age or younger.

	**Percutaneous device closure**				**Surgery**				**All ASD interventions**			
	**Univariate**		**Adjusted**		**Univariate**		**Adjusted**		**Univariate**		**Adjusted**	
	**0R**	**95% C.I**.	**0R**	**95% C.I**.	**0R**	**95% C.I**.	**0R**	**95% C.I**.	**0R**	**95% C.I**.	**0R**	**95% C.I**.
Gender (female)	0.8	0.4–1.4	−	−	1.6	0.8–3.1			1.1	0.7–1.7		
Preterm	2.2	1.2–4.2	2.1	0.9–4.6	2.9	1.3–6.3	1.8	0.5–6.8	2.4	1.5–3.9	1.7	0.9–3.3
Late preterm	1.8	0.9–3.5	−	−	2.1	0.8–5.3			1.8	1.0–3.1		
Very Preterm	4.6	1.2–16.8	−	−	x	x			6.4	2.0–20.4		
Extremely preterm	1.1	0.2–6.0	−	−	1.4	0.3–5.9			1.5	0.5–4.2		
<32 gestational weeks	1.0	1.0–7.4	4.2	0.3–61.0	2.6	0.8–7.5	0.5	0.1–9.3	3.0	1.5–6.0	2.0	0.5–9.0
Very low birthweight	2.4	0.8–6.6	0.2	0.1–3.0	2.1	0.6–6.9	1.6	0.1–32.7	2.3	1.1–5.0	0.4	0.1–1.9
Extremely low birthweight	2.2	0.5–10.1	−	−	1.4	0.3–6.0			2.0	0.7–5.5		
Chromosomal abnormalities	3.6	1.7–8.0	3.2	1.4–7.5	3.5	1.2–10.7	2.1	0.5–9.5	3.4	1.8–6.5	2.7	1.4–5.4
Other CHD	1.2	0.6–2.2	0.8	0.4–1.7	6.5	3.1–13.7	4.4	1.9–10.5	2.6	1.7–4.1	1.9	1.1–3.2
Arrhythmias before closure	1.8	0.4–7.6			0.5	0.1–2.9			1.1	0.4–3.2		
Infant respiratory distress syndrome	1.4	0.5–4.1			4.5	0.9–22.9			1.9	0.9–4.3		
Bronchopulmonary dysplasia	1.2	0.3–4.9			3.7	0.7–20.0			1.9	0.7–5.2		
Neonatal ventilatory support	3.6	1.1–12.2	1.3	0.2–7.9	1.4	0.4–5.1	0.1	0.1–0.4	2.5	1.0–5.9	0.3	0.1–1.3
Neonatal CPAP^*^	2.2	0.9–4.9	0.8	0.2–3.0	4.2	1.5–11.6	4.4	0.6–33.3	2.9	1.6–5.3	1.6	0.6–3.9
Neonatal sepsis	2.7	0.9–7.6	1.7	0.5–6.0	5.5	1.1–27.3	12.5	0.6–264	3.3	1.4–7.6	2.4	0.8–7.1
Pulmonary hypertension	5.0	1.9–13.0	3.9	1.2–12.1	9.1	1.9–42.66	13.2	1.2–143	5.8	2.6–12.6	3.7	1.5–9.2

Univariate analysis.

* *Continuous Positive Airway Pressure*.

In a univariate analysis, very and extremely premature birth were also associated with the risk of an even earlier ASD closure, at 1 year of age and younger, for all used ASD closure methods (Table 4). All neonatal morbidities and medical support, as well as additional congenital and chromosomal abnormalities, were associated with ASD closure at 1 year of age or younger (Table 4).

**Table 4:** Risk factors for ASD closure at 1 year of age or younger.

	**All ASD closures**	
	**0R**	**95% C.I**.
Late preterm	1.1	0.5–2.4
Very preterm	9.7	3.3–28.1
Extremely preterm	5.4	1.8–15.8
<32 gestational weeks	6.9	3.3–14.8
Very low birthweight	5.8	2.5–13.3
Extremely low birthweight	5.4	1.8–15.9
Chromosomal abnormalities	3.6	1.7–7.5
Other congenital heart defect	4.7	2.6–8.7
Infant respiratory distress syndrome	4.5	1.9–10.6
Bronchopulmonary dysplasia	4.4	1.5–12.3
Neonatal ventilatory support	6.3	2.5–16.0
Neonatal CPAP^*^	6.5	3.3–12.8
Neonatal sepsis	6.4	2.7–15.3
Pulmonary hypertension	11.1	5.1–24.0

Univariate analysis.

* *Continuous Positive Airway Pressure*.

The risk of requiring early ASD percutaneous device closure was associated with additional chromosomal abnormalities, OR = 3.2 (95% CI: 1.4–7.5); pulmonary hypertension, OR = 3.9 (95% CI:1.2–12.1); additional CHD, OR = 4.4 (95% CI: 1.9–10.5); and pulmonary hypertension, OR = 13.2 (95% CI: 1.2–143), after adjustments were made for confounding factors (Table 5). An ASD size:body weight ratio of 2.0, as well as a ratio of 0.8, was associated with increased risk of an early ASD closure, even after adjustments for confounding factors were made (Table 5).

**Table 5:** Adjusted risk factors for early closure in relation to ASD size and body weight ratio.

	**Percutaneous device closure**		**Surgical closure**		**All ASD interventions**	
	**0R**	**95% C.I**.	**0R**	**95% C.I**.	**0R**	**95% C.I**.
**ASD size: body weight ratio = 2.0**						
ASD size:body weight ratio = 2.0	9.9	1.0–95.5	21.4	1.9–236.0	21.0	4.31–102.0
Preterm birth (<37GW)	6.1	0.7–53.0	1.3	0.1–31.4	1.9	1.0–3.7
Chromosomal abnormalities	2.0	1.3–7.5	1.8	0.4–8.7	2.4	1.2–5.0
Other congenital heart defects	0.8	0.4–1.8	4.6	1.9–11.3	1.9	1.1–3.2
Pulmonary hypertension	3.1	0.9–10.3	6.1	0.5–68.3	2.7	1.0–7.2
**ASD size:body weight ratio = 0.8**						
ASD size:body weight ratio = 0.8	8.9	4.3–18.5	5.4	2.0–14.3	7.06	4.39–13.18
Preterm birth (<37GW)	1.5	0.6–3.7	3.4	0.8–14.0	1.7	0.8–3.6
Chromosomal abnormalities	2.9	1.1–7.5	1.1	0.2–5.7	2.0	0.9–4.4
Other congenital heart defects	0.9	0.4–2.2	4.0	1.6–9.9	1.9	1.1–3.3
Pulmonary hypertension	4.1	1.1–15.1	11.5	1.0–129.4	4.1	1.5–11.3

*Multivariate analysis*.

## Discussion

An atrial septal defect (ASD) allows the shunting of blood from the systemic and pulmonary circulation and causes right heart volume overload (15). Spontaneous closure has been described for small or moderate sized ASD (16–18). In children with an asymptomatic ASD, guidelines recommend elective ASD closure at 3to 5 years old (15). Closure of ASD has been suggested when there is clinical evidence of volume overload with pulmonary to systemic blood flow (Qp:Qs) >1.5 and right heart enlargement. ASD-related symptoms, such as failure to thrive and impaired exercise tolerance, pulmonary hypertension and failure to wean from respiratory support may lead to early ASD closure (1, 15, 19, 20). In our study, persistent pulmonary hypertension after birth was linked to an increased risk of early closure, especially when the ASD size: weight ratio was low. Thus, recommendations for closure, such as pulmonary hypertension, were met in our study. An Qp:Qs >1.5, as well as right heart enlargement, was equally common among cases and controls, indicating that the risk of overtreatment among cases is limited in our study. The risk of selection bias due overtreatment of young children in our study was thus limited.

### Premature Birth

Children born prior to a gestational age of 37 weeks (premature), have an altered myocardium compared to term-born children (5–7, 21). These alterations, as described in echocardiographic and magnetic resonance imaging studies, are present even long after the neonatal period (6–10, 21). Preterm birth (prior to 32 gestational weeks), as well an extremely preterm birth (prior to 28 gestational weeks), was more common among children with an early closure before 2 years of age, as well as for closure before 1 year of age. However, the association between an early closure and premature birth was only present when adjustments for confounding factors were made, including ASD size:body weight ratio = 2. Thus, children with a low bodyweight at closure, as is common in preterm children, and with a larger ASD size, have an increased risk of ASD closure at young age. The myocardial impairment recently found in prematurely born children, perhaps along with smaller heart volumes, can have an impact on this risk.

### Surgical Repair

Spontaneous closure has been described in ASDs of 5 mm or less. However, some ASDs may enlarge over time (18, 19). Numerous studies have described the safety and efficacy of ASD closure, and it is suggested that the percutaneous approach is preferable to surgery in most patients (20, 22, 23). In our study, the presence of other congenital heart defects was associated with an increased risk of ASD closure. This risk was, not surprisingly, greater among the surgical closures (OR = 4.4 (95% CI 1.9–10.5), and can be explained by the ASD being closed at the same time as other, more severe heart defects, were repaired. This risk remained unaltered even after adjustments, as well as for children with an ASD size:body weight ratio = 2 and especially an ASD size:body weight ratio = 0.8. Therefore, in cases where a surgical repair was needed for other cardiac diseases, the ASD was closed even when the ASD size was small and bodyweight increased (ASD size:body weight ratio = 0.8). The surgeons are thus not likely to leave an ASD even if there could be a chance of spontaneous closure.

### ASD Size:Bodyweight Ratio

According to current guidelines, a hemodynamically significant ASD with enlarged right side heart structures should be closed electively once the diagnosis is confirmed (1). Factors such as increased ASD size:bodyweight ratio have previously been described as predicting the risk of complications following ASD device closure (24, 25). An ASD size:bodyweight ratio = 1.2 or less has been considered optimal for percutaneous device closure (24–26). In our study, the risk of an early ASD closure was associated with estimated ASD size:bodyweight ratio of 0.8 and of 2.0, even after adjustments for potential confounders. In a previous study, we did not find an increased risk of adverse events following ASD closure for children with large or small ASD size:bodyweight ratios (13). This indicates that the ASD size:bodyweight ratio cannot be the only predictor of the need for an early closure nor for the risk of adverse events.

### Risk Factors for Early Closure

It is well-known that congenital heart defects are more common among children with chromosomal abnormalities. Down syndrome is known to increase the risk of pulmonary hypertension, as well as symptoms of volume overload and heart failure in the presence of shunt defects (27–29). Chromosomal abnormalities were associated with an increased risk of an early ASD closure in our study, OR = 3.2 (95% CI: 1.4–7.5). This risk was present for surgical, as well as device, closure, even when other factors were accounted for. Several studies have shown ASD repair in children with Down syndrome to be beneficial and safe (28, 30). Children with chromosomal abnormalities may have earlier signs of enlarged right side heart structures and symptoms caused by volume overload. This study indicates that children with chromosomal abnormalities might be more sensitive to volume overload.

The cases in our study, children at 2 years of age or younger at closure, as well as 1 year of age and younger, obviously had a lower bodyweight than the controls of an older age. The benefits of ASD closure are well studied, and complete resolution of right ventricular enlargements have been described (31, 32). The risk of adverse effects during and following ASD closures has also been studied for small children, even those born prematurely, and it is considered safe and effective (2, 4, 23). A relationship between an improved myocardial function and an early closure of an atrial septal defect, especially in prematurely born children, has yet to be studied. The indications and predictors of an early closure have to be considered in these future studies.

### Limitations and Strengths

This case-control study used medical records, as well as national registries to retrieve data. The risk of selection and recall bias is limited through using several sources. The registries have been validated, with good coherence between the registries and medical records (33, 34). Two out of three Swedish heart centers that perform percutaneous device closure of ASD and one of two operating centers in Sweden contributed data to our study. This reduces selection bias and increases the number of included patients, which increases the power of the study (35).

## Conclusions

An increased risk of an early ASD closure is associated with additional chromosomal abnormalities and pulmonary hypertension. A larger ASD size in relation to lighter bodyweight (ratio = 2), or smaller ASD size in relation to a heavier bodyweight (ratio = 0.8) were associated with increased risk of an early ASD closure. The ratio between ASD size and bodyweight has a wide range when it comes to predicting an early ASD closure and might not be the best indicator of the need for ASD closure.

## Data Availability Statement

The datasets generated for this study are available on request to the corresponding author.

## Ethics Statement

The authors assert that all procedures contributing to this work comply with the ethical standards of the Helsinki Declaration of 1975, as revised in 2008. This study was approved by the Ethics Committee for Human Research at the Umeå University (D-nr 2015-10-31M alteration 2015-88-32M), and informed consent was obtained by everyone in the study population or each guardian for the included children.

## Author Contributions

GT performed the data analysis. GT, PL, and GS participated in the analytical framework and contributed to the writing of the manuscript. EN had primary responsibility for study, protocol development, patient enrolment, outcome assessment, participated in the analytical framework, and had the primary responsibility for writing the manuscript.

### Conflict of Interest

The authors declare that the research was conducted in the absence of any commercial or financial relationships that could be construed as a potential conflict of interest.
